# Diphtheria in Mayotte, 2007–2015

**DOI:** 10.3201/eid2307.170262

**Published:** 2017-07

**Authors:** Emmanuel Belchior, Sabine Henry, Edgar Badell, Louis Collet, Thierry Benoit-Cattin, Anne-Marie de Montera, Nicole Guiso, Olivier Patey, Daniel Levy-Bruhl, Laurent Filleul, Francois Chieze, Sophie Olivier

**Affiliations:** Santé Publique France, Saint-Maurice, France (E. Belchior, D. Levy-Bruhl);; Agence Régionale de Santé Océan Indien, Saint-Denis, France (S. Henry, F. Chieze);; Institut Pasteur, Paris, France (E. Badell, N. Guiso);; Centre Hospitalier de Mayotte, Mamoudzou, France (L. Collet, T. Benoit-Cattin, A.-M. de Montera, S. Olivier);; Centre Hospitalier Intercommunal, Villeneuve-Saint-Georges, France (O. Patey);; Santé Publique France, Saint-Denis de La Réunion, France (L. Filleul)

**Keywords:** diphtheria, Corynebacterium diphtheriae, bacteria, epidemiology, toxigenic, vaccination, southwestern Indian Ocean region, Mayotte, France

## Abstract

Epidemiology of diphtheria in the southwestern Indian Ocean is poorly documented. We analyzed 14 cases of infection with toxigenic *Corynebacterium diphtheriae* reported during 2007–2015 in Mayotte, a French department located in this region. Local control of diphtheria is needed to minimize the risk for importation of the bacterium into disease-free areas.

Diphtheria due to toxigenic *Corynebacterium diphtheriae* occurs sporadically across Europe, mostly in persons who emigrated from disease-endemic countries (*1*,*2*). Systemic toxic effects occur in cutaneous diphtheria but less commonly than in pharyngeal or laryngeal diphtheria. *C. diphtheriae* is a well-recognized cause of chronic, nonhealing skin ulcers in the tropics (*3*,*4*). In France, diphtheria has been a reportable disease since 1945. A vaccination program began in the late 1940s; no cases were reported during 1990–2001, and 13 cases were reported, all in visitors or immigrants to France, since 2002. However, the epidemiology of diphtheria in the southwestern Indian Ocean region, where several French departments are located, is poorly documented.

Mayotte, a French department, is an island of the Comoros archipelago (Figure) and an attractive destination for migrants from the area. In 2012, Mayotte had 212,645 inhabitants, at least 40% foreign born (*5*). We analyzed all cases of infection with toxigenic *C. diphtheriae* reported in Mayotte since 2007 to evaluate the potential risk for dissemination of diphtheria inside and outside this area.

**Figure:**
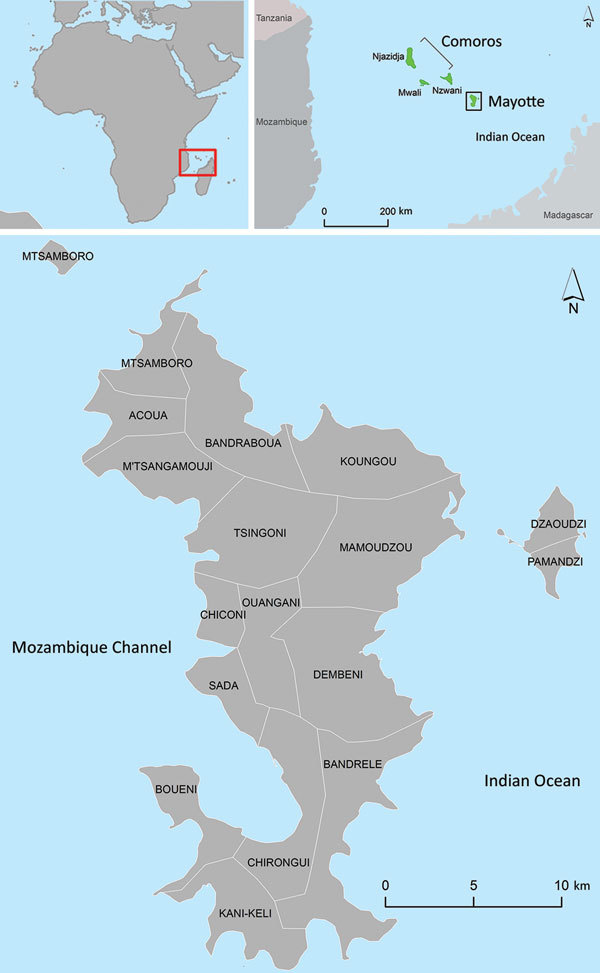
Location of Mayotte in the southwestern Indian Ocean. Maps created by using IGN-GEOFLA (http://professionnels.ign.fr/geofla) and Esri Data and Maps 10 (http://www.esri.com/).

During 2007–2015, local hospitals reported 14 cases of toxigenic *C. diphtheriae* infection to the local health authorities: 1 case in an infant with severe respiratory symptoms who died from multiple organ failure and 13 cutaneous diphtheria cases in patients who survived. Most of the patients (11/14) were male, and the median age was 11 years (range 2 months–39 years). Eight patients had recently (most within 1 month) emigrated from neighboring islands.

Patients’ medical history was usually unknown, but vaccination status was available for 12 patients: 7 had received >3 doses of diphtheria vaccine according to the vaccination schedule of France (*6*) and 5 were unvaccinated (the infant, 2 children, and 2 adults). All cutaneous infections occurred in patients with preexisting wounds. Eleven patients had chronic diphtheria infections (1–36 months) with co-infections of other pathogens such as *Staphylococcus aureus* and *Streptococcus pyogenes*. All patients received antimicrobial drug therapy with surgical debridement if necessary. In the absence of signs of toxin dissemination, none of the patients with cutaneous diphtheria received diphtheria antitoxin. 

Diagnosis was established by isolation of *C. diphtheriae* and PCR detection of the toxin gene. All 14 isolates were sent to the National Reference Centre (Paris, France) to confirm the identification of the clinical isolates (all positive for *C. diphtheriae tox* gene), determine their biotypes (2 variant *gravis* and 12 variant *mitis*), and characterize their toxin production using the Elek test (positive for 9 isolates). All isolates were sensitive to a large spectrum of antimicrobial drugs, except for fosfomycin. All investigations were conducted by local health authorities to implement control measures (throat swab specimens and antimicrobial prophylaxis of close contacts), according to national recommendations (*7*). Among 168 identified close contacts, we observed 8 asymptomatic respiratory carriers of toxigenic *C. diphtheriae*.

Poor health status and substandard living conditions are risk factors associated with transmission of diphtheria. Cutaneous diphtheria has been shown to be more contagious than respiratory diphtheria (*8*). Furthermore, cutaneous diphtheria may be clinically indistinguishable from other common skin lesions in the tropics and underdiagnosed in cases of co-infection or confusion with the normal skin flora.

Due to its geographic location and given its low socioeconomic status, the population of Mayotte remains exposed to diphtheria. Absence of widespread circulation of toxigenic *C. diphtheriae* cannot be ruled out because of living conditions, the population’s difficulties in accessing care, frequency of skin infections, and complexity of microbiological analyses of cutaneous samples. The high coverage for diphtheria vaccination in young children as estimated in a 2010 survey (95% for those 24–59 months of age) is likely to have contributed to the absence of respiratory cases and of systemic complications (*9*). Another possible explanation is that a high rate of skin infections caused by *C. diphtheriae* may result in early development or boosting of natural immunity against the disease (*10*).

Several elements are in favor of a persisting risk: pockets of nonvaccinated populations (many children and teenagers and most adults) in the local context of illegal immigration from neighboring countries pose a risk of severe disease and of persistence of bacterium transmission. The finding that several isolates were producing the toxin is another element, as is the possibility of transmission from a cutaneous lesion to the throat of a close contact. Of note, >20 nontoxigenic isolates (in which PCRs did not detect the *tox* gene) were collected from cutaneous wounds during the same period. In accordance with national guidelines, no control measures were implemented around these cases.

Because human migrations across the area seem inevitable, improving sanitary conditions such as access to clean drinking water and maintaining high immunity levels in the population through vaccination are critical for local prevention and control of diphtheria. Use of these tools will minimize the risk of importation of *C. diphtheriae* into disease-free areas.

## References

[1] Wagner KS, White JM, Neal S, Crowcroft NS, Kuprevičiene N, Paberza R, et al.; Members of the Diphtheria Surveillance Network. Screening for *Corynebacterium diphtheriae* and *Corynebacterium ulcerans* in patients with upper respiratory tract infections 2007-2008: a multicentre European study. Clin Microbiol Infect. 2011;17:519–25. 10.1111/j.1469-0691.2010.03269.x 20491827

[2] Meinel DM, Kuehl R, Zbinden R, Boskova V, Garzoni C, Fadini D, et al. Outbreak investigation for toxigenic *Corynebacterium diphtheriae* wound infections in refugees from Northeast Africa and Syria in Switzerland and Germany by whole genome sequencing. Clin Microbiol Infect. 2016;22:1003.e1–8. 10.1016/j.cmi.2016.08.010 27585943

[3] Belsey MA, LeBlanc DR. Skin infections and the epidemiology of diphtheria: acquisition and persistence of C diphtheriae infections. Am J Epidemiol. 1975;102:179–84. 10.1093/oxfordjournals.aje.a112145 808123

[4] Thaung U, Naung T, Saw Khine K, Khai Ming C. Epidemiological features of skin diphtheria infection in Rangoon, Burma. Southeast Asian J Trop Med Public Health. 1978;9:4–10. 151920

[5] Institut National de la Statistique et des Etudes Economiques (Insee). Mayotte, France's youngest department. [cited 2017 May 3]. https://www.insee.fr/en/statistiques/1281385

[6] French Vaccination Schedule [in French] [cited 2017 May 3]. http://social-sante.gouv.fr/IMG/pdf/calendrier_vaccinal_2016.pdf

[7] Instruction DGS/RI1 no 2011–348 du 30 août 2011 relative à la conduite à tenir lors de l’apparition d’un cas de diphtérie [cited 2017 May 3]. http://circulaire.legifrance.gouv.fr/pdf/2011/09/cir_33827.pdf

[8] Koopman JS, Campbell J. The role of cutaneous diphtheria infections in a diphtheria epidemic. J Infect Dis. 1975;131:239–44. 10.1093/infdis/131.3.239 805182

[9] Solet JL. Enquête de couverture vaccinale à Mayotte en 2010. Saint-Maurice: Institut de veille sanitaire; 2012 [cited 2017 May 3]. http://invs.santepubliquefrance.fr/Publications-et-outils/Rapports-et-syntheses/Maladies-infectieuses/2012/Enquete-de-couverture-vaccinale-a-Mayotte-en-2010

[10] Expanded Programme on immunization. The immunological basis for immunization series module 2: diphtheria. Geneva: World Health Organization, 1993. WHO/EPI/GEN/93.12 [cited 2017 May 3]. http://www.nccvmtc.org/pdf1/1_028.pdf

